# Prevalence and clinico-genomic characteristics of patients with TRK fusion cancer in China

**DOI:** 10.1038/s41698-023-00427-3

**Published:** 2023-08-11

**Authors:** Yujun Xu, Xiaoliang Shi, Weifeng Wang, Lin Zhang, Shinghu Cheung, Marion Rudolph, Nicoletta Brega, Xiaowei Dong, Lili Qian, Liwei Wang, Shaohua Yuan, Daniel Shao Weng Tan, Kai Wang

**Affiliations:** 1 Department of Imaging Interventional Therapy, Shandong Provincial Hospital, Cheeloo College of Medicine, Shandong University; Department of Imaging Interventional Therapy, Shandong Provincial Hospital Affiliated to Shandong First Medical University, 250021 Jinan, China; 2 OrigiMed Co. Ltd, 201114 Shanghai, China; 3 Precision Molecular Oncology, Research and Early Development - Oncology, Pharmaceuticals, Bayer U.S. LLC, Cambridge, USA; 4 Translational Sciences Oncology, Research and Early Development - Oncology, Pharmaceuticals, Bayer AG, Berlin, Germany; 5 Strategic Business Unit Oncology, Bayer S.p.A, Milan, Italy; 6 National Cancer Centre Singapore, Duke-NUS Medical School, 169610 Singapore, Singapore

**Keywords:** Tumour biomarkers, Molecular medicine

## Abstract

Neurotrophic tyrosine kinase (*NTRK*) fusions involving *NTRK1*, *NTRK2*, and *NTRK3* were found in a broad range of solid tumors as driver gene variants. However, the prevalence of *NTRK* fusions in Chinese solid tumor patients is rarely reported. Based on the next-generation sequencing data from 10,194 Chinese solid tumor patients, we identified approximately 0.4% (40/10,194) of Chinese solid tumor patients with *NTRK* fusion. *NTRK* fusions were most frequently detected in soft tissue sarcoma (3.0%), especially in the fibrosarcoma subtype (12.7%). A total of 29 *NTRK* fusion patterns were identified, of which 11 were rarely reported. *NTRK* fusion mostly co-occurred with *TP53* (38%), *CDKN2A* (23%), and *ACVR2A* (18%) and rarely with *NTRK* amplification (5.0%) and single nucleotide variants (2.5%). DNA-based *NTRK* fusion sequencing exhibited a higher detection rate than pan-TRK immunohistochemistry (100% vs. 87.5%). Two patients with *NTRK* fusions showed clinical responses to larotrectinib, supporting the effective response of *NTRK* fusion patients to TRK inhibitors.

## Introduction

Neurotrophic tyrosine kinase (*NTRK*) genes, involving *NTRK1, NTRK2*, and *NTRK3*, encode the three transmembrane proteins, tropomyosin receptor kinase (TRK) A, B, and C of the TRK family, which are the receptors of nerve growth factor (NGF), brain-derived growth factor (BDNF), and neurotrophin 3 (NTF-3), respectively^1,2^. TRK receptors play important roles in the development and homeostasis of the nervous system. The *NTRK1, NTRK2*, and *NTRK3* genes are located on chromosomes 1q23.1, 9q22.1, and 15q25, respectively^3^.

*NTRK* fusions caused by interchromosomal or intrachromosomal rearrangement represent functional genomic alterations in many cancers. The chimeric oncoprotein containing the TRK tyrosine kinase domain is constitutively activated and/or overexpressed, resulting in the activation of downstream pro-oncogenic pathways^4^. *NTRK* fusions were found in about 0.3% of solid tumors, and the frequency of fusion was different by cancer types^5^. *NTRK* fusions have recently been identified as targets for cancer therapy as well^6^, culminating in the first global approval by the Food and Drug Administration (FDA) for Larotrectinib in November 2018, followed by approval in more than 45 countries, including China, for the treatment of adult and pediatric patients with metastatic or locally advanced solid tumors harboring an *NTRK* fusion gene. Notably, due to the broad durable activity across a range of cancer types, this was the first approval of tissue agnostic drug for any solid tumors harboring an *NTRK* fusion^7^.

More than 80 partner genes have been reported to fuse with three *NTRK* genes, generating even more chimeric proteins^8^. Therefore, the classic assays for gene rearrangement, such as fluorescent in situ hybridization (FISH) and reverse transcription polymerase chain reaction (RT-PCR), are incompetent for the detection of complex *NTRK* fusions, such as different fusion partners and breakpoints. Next-generation sequencing (NGS)-based detections, particularly those utilizing RNA as a starting material, are recommended for *NTRK* fusion detection^9–11^.

Exploration of the incidence of *NTRK* fusions in a variety of solid tumors is limited^12^; in particular, data from a large cohort of Chinese patients with solid tumors has not been reported. In this study, we detected and comprehensively characterized the prevalence of *NTRK* fusions in more than 10,000 Chinese patients with solid tumors and further investigated whether NGS is a reliable method for *NTRK* fusion detection in clinical practice.

## Results

### Patient characteristics

The clinical characteristics of 10,194 patients enrolled in this study are summarized in Table 1. The cohort (Supplementary Fig. 1, Table 1) included non-small cell lung cancer (*N* = 2039, 20.0%), colorectal cancer (*N* = 1225, 12.0%), hepatocellular carcinoma (*N* = 1133, 11.1%), gastric cancer (*N* = 866, 8.5%), esophageal carcinoma (*N* = 610, 6.0%), soft tissue sarcoma (*N* = 571, 5.6%), intrahepatic cholangiocarcinoma (*N* = 555, 5.4%), pancreatic cancer (*N* = 498, 4.9%), extrahepatic cholangiocarcinoma (*N* = 351, 3.4%), breast cancer (*N* = 323, 3.2%), renal cell carcinoma (*N* = 308, 3.0%), ovarian cancer (*N* = 261, 2.6%), gallbladder carcinoma (*N* = 240, 2.4%), small cell lung cancer (*N* = 220, 2.2%), bone sarcoma (*N* = 183, 1.8%), head and neck carcinoma (*N* = 175, 1.7%), cancer of unknown primary (*N* = 120, 1.2%), endocervical carcinoma (*N* = 104, 1.0%), and others (*N* = 412, 4.0%). The median age of patients was 58 years, ranging from ≤1 to 96 years. In the cohort, 60.4% were male and 39.6% were female.

**Table 1:** The clinical characteristics in all *NTRK* fusion-positive and *NTRK* fusion-negative cohorts.

	Overall (*N* = 10,194)	*NTRK* fusion-positive (*N* = 40)	*NTRK* fusion-negative (*N* = 10,154)	*P*-value
Age, median (range), years	58.0 [1.0, 96.0]	45.0 [≤1.0, 76.0]	58.0 [1.00, 96.0]	<0.001
Infantile (≤1), *n* (%)	12 (0.1%)	4 (10.0%)	8 (0.1%)	
Pediatric (>1 to ≤18), *n* (%)	157 (1.5%)	6 (15.0%)	151 (1.5%)	
Adult (>18), *n* (%)	10,025 (98.3%)	30 (75.0%)	9995 (98.4%)	
Gender				0.79
Female	4039 (39.6%)	15 (37.5%)	4024 (39.6%)	
Male	6155 (60.4%)	25 (62.5.0%)	6130 (60.4%)	
Tumor stage, *n* (%)				0.64
I-II	3571 (35%)	11 (27.5%)	3560 (35.1%)	
III-IV	5652 (55.4%)	23 (57.5%)	5629 (55.4%)	
Unknown	971 (9.5%)	6 (15.0%)	965 (9.5%)	
TMB, median (range), muts/Mb	4.60 [0.00, 825]	3.80 [0.0, 180]	4.60 [0.00, 825]	0.19
MSI status, *n* (%)				<0.001
MSI-H	186 (1.8%)	6 (15.0%)	180 (1.8%)	
MSS	9461 (92.8%)	32 (80.0%)	9429 (92.9%)	
Unknown	547 (5.4%)	2 (5.0%)	545 (5.4%)	
Tumor_type, *n* (%)				<0.001
Soft tissue sarcoma	571 (5.6%)	17 (45.5%)	554 (5.5%)	
Colorectal cancer	1225 (12.0%)	7 (17.5%)	1218 (12.0%)	
Non-small cell lung cancer	2039 (20.0%)	5 (12.5%)	2034 (20.0%)	
Hepatocellular carcinoma	1133 (11.1%)	2 (5.0%)	1131 (11.1%)	
Breast cancer	323 (3.2%)	1 (2.5%)	322 (3.2%)	
Small cell lung cancer	220 (2.2%)	2 (5.0%)	218 (2.1%)	
Extrahepatic cholangiocarcinoma	351 (3.4%)	1 (2.5%)	350 (3.4%)	
Head and neck carcinoma	175 (1.7%)	1 (2.5%)	174 (1.7%)	
Intrahepatic cholangiocarcinoma	555 (5.4%)	1 (2.5%)	554 (5.5%)	
Ovarian cancer	261 (2.6%)	1 (2.5%)	260 (2.6%)	
Gastric cancer	866 (8.5%)	1 (2.5%)	865 (8.5%)	
Thyroid tumor	32 (0.3%)	1 (2.5%)	31 (0.3%)	
Gallbladder carcinoma	240 (2.4%)	0 (0%)	240 (2.4%)	
Bone sarcoma	183 (1.8%)	0 (0%)	183 (1.8%)	
Cancer of unknown primary	120 (1.2%)	0 (0%)	120 (1.2%)	
Endocervical carcinoma	104 (1.0%)	0 (0%)	104 (1.0%)	
Endometrial carcinoma	61 (0.6%)	0 (0%)	61 (0.6%)	
Esophageal carcinoma	610 (6.0%)	0 (0%)	610 (6.0%)	
Gastrointestinal neuroendocrine tumor	74 (0.7%)	0 (0%)	74 (0.7%)	
Melanoma	59 (0.6%)	0 (0%)	59 (0.6%)	
Pancreatic cancer	498 (4.9%)	0 (0%)	498 (4.9%)	
Renal cell carcinoma	308 (3.0%)	0 (0%)	308 (3.0%)	
Small bowel carcinoma	57 (0.6%)	0 (0%)	57 (0.6%)	
Thymic tumor	33 (0.3%)	0 (0%)	33 (0.3%)	
Urothelial carcinoma	96 (0.9%)	0 (0%)	96 (0.9%)	

*P*-values are calculated by the Wilcox test or Fisher test among *NTRK* fusion-positive and *NTRK* fusion-negative patients, *P* < 0.01 represented the significant difference.

In this cohort, there were 40 patients with *NTRK* fusion, 13 of which underwent multiple detection methods, including DNA-seq, RNA-seq, and immunohistochemistry (IHC). There was a significant difference in the age distribution between *NTRK* fusion-positive (*N* = 40, 0.4%) and *NTRK* fusion-negative (*N* = 10,154) patients (median age, 45 vs. 58 years, respectively, *P* < 0.01).

Among 40 patients with *NTRK* fusions, 4 cases arose in infants (age ≤ 1 year), 6 in pediatric patients (age range from 1 to 18 years), and 30 in adult patients (age > 18 years). Among 10 *NTRK* fusion-positive infantile/pediatric patients (age ≤ 18 years), there were 10 soft tissue sarcomas, including 6 infantile fibrosarcomas, 1 cellular plexiform schwannoma, 1 rhabdomyosarcoma, and 2 soft tissue sarcomas of the abdomen (Tables 1 and 2). However, there was no apparent difference in sex and tumor stage distribution between *NTRK* fusion-positive and fusion-negative patients. Although tumor mutational burden (TMB) value was comparable between these two cohorts (*NTRK* fusion-positive vs. *NTRK* fusion-negative: 4.6 [0–180] vs. 4.6 [0–825] muts/Mb, *P* = 0.19), *NTRK* fusions were enriched in tumors with high microsatellite instability (MSI-H) (MSI-H vs. microsatellite stability [MSS]: 3.2% [6/186] vs. 0.34% [32/9461]; *P* < 0.001), including 4 colorectal cancers, 1 gastric cancer, and 1 intrahepatic cholangiocarcinoma (Tables 1 and 2).

**Table 2:** The clinicopathologic and molecular characteristics of 40 patients harboring *NTRK* fusions.

Case	Age (years)	Gender	Histologic diagnosis	Subtype	NTRK fusions	Fusion classification	Fusion type	TMB (muts/Mb)	MSI status	Fusion novelty
1	1	Female	Soft tissue sarcoma	Infantile fibrosarcoma	*ETV6-NTRK3*	Definite fusion	Interchromosomal rearrangement	0	MSS	Reported
2	1	Male	Soft tissue sarcoma	Infantile fibrosarcoma	*ETV6-NTRK3*	Definite fusion	Interchromosomal rearrangement	0	MSS	Reported
3	1	Female	Soft tissue sarcoma	Infantile fibrosarcoma	*ETV6-NTRK3*	Definite fusion	Interchromosomal rearrangement	6.9	MSS	Reported
4	1	Female	Soft tissue sarcoma	Infantile fibrosarcoma	*ETV6-NTRK3*	Definite fusion	Interchromosomal rearrangement	0.8	MSS	Reported
5	2	Female	Soft tissue sarcoma	Cellular plexiform schwannoma	*TPM3-NTRK1*	Definite fusion	Intrachromosomal rearrangement	0	MSS	Reported
6	2	Male	Soft tissue sarcoma	Infantile fibrosarcoma	*ETV6-NTRK3*	Definite fusion	Interchromosomal rearrangement	3	MSS	Reported
7	3	Male	Soft tissue sarcoma	Rhabdomyosarcoma	*LMNA-NTRK1*	Definite fusion	Intrachromosomal rearrangement	1.2	MSS	Reported
8	4	Male	Soft tissue sarcoma	Infantile fibrosarcoma	*TPM3-NTRK1*	Definite fusion	Intrachromosomal rearrangement	0.7	MSS	Reported
9	9	Male	Soft tissue sarcoma	Soft tissue sarcoma of the abdomen	*LMNA-NTRK*1	Definite fusion	Intrachromosomal rearrangement	1.2	MSS	Reported
10	15	Male	Soft tissue sarcoma	Soft tissue sarcoma of the abdomen	*LMNA-NTRK1*	Definite fusion	Intrachromosomal rearrangement	0.6	MSS	Reported
11	23	Male	Carcinoma	Mucoepidermoid carcinoma of trachea	*C7orf69-NTRK*3	Likely fusion	Interchromosomal rearrangement	0	MSS	Novel
12	29	Male	Soft tissue sarcoma	Spindle cell sarcoma of the prostate	*RBPMS-NTRK3*	Definite fusion	Interchromosomal rearrangement	1.5	MSS	Reported
13	35	Male	Soft tissue sarcoma	Prostatic stromal tumor	*IRF2BP2-NTRK1*	Definite fusion	Intrachromosomal rearrangement	4.7	Unknown	Reported
14	38	Female	Carcinoma	Thyroid tumor	*ETV6-NTRK3*	Definite fusion	Interchromosomal rearrangement	1.5	MSS	Reported
15	42	Female	Soft tissue sarcoma	Spindle cell sarcoma of the thigh	*LMNA-NTRK1*	Definite fusion	Intrachromosomal rearrangement	0.7	MSS	Reported
16	42	Male	Soft tissue sarcoma	Mucinous liposarcoma	*MORF4L1-NTRK3*	Definite fusion	Intrachromosomal rearrangement	0.7	MSS	Novel
16	42	Male	Soft tissue sarcoma	Mucinous liposarcoma	*PPFIA2-NTRK3*	Definite fusion	Interchromosomal rearrangement	0.7	MSS	Novel
17	43	Male	Soft tissue sarcoma	Spindle cell tumors of the sacrum	*LMNA-NTRK1*	Definite fusion	Intrachromosomal rearrangement	3	MSS	Reported
18	43	Female	Carcinoma	Non-small cell lung cancer	*TPM3-NTRK1*	Definite fusion	Intrachromosomal rearrangement	0.8	MSS	Reported
19	43	Male	Carcinoma	Small cell lung cancer	*ETV6-NTRK3*	Definite fusion	Interchromosomal rearrangement	16.4	MSS	Reported
20	43	Male	Carcinoma	Gastric cancer	*INSRR-NTRK1*	Likely fusion	Intrachromosomal rearrangement	44.1	MSI-H	Novel
21	44	Male	Soft tissue sarcoma	High-grade spindle cell sarcoma of the small intestine	*LMNA-NTRK1*	Definite fusion	Intrachromosomal rearrangement	2.5	MSS	Reported
22	46	Male	Carcinoma	Intrahepatic cholangiocarcinoma	*PHF20-NTRK1*	Likely fusion	Interchromosomal rearrangement	42.5	MSI-H	Reported
23	48	Female	Carcinoma	Small cell lung cancer	*ETV6-NTRK3*	Definite fusion	Interchromosomal rearrangement	14.8	MSS	Reported
24	48	Female	Carcinoma	Non-small cell lung cancer	*RB1-NTRK3*	Definite fusion	Interchromosomal rearrangement	5.4	MSS	Novel
25	48	Male	Carcinoma	Hepatocellular carcinoma	*TTC28-NTRK3*	Likely fusion	Interchromosomal rearrangement	6.9	MSS	Novel
26	52	Male	Carcinoma	Non-small cell lung cancer	*AMOTL2-NTRK1*	Likely fusion	Interchromosomal rearrangement	8.7	Unknown	Reported
27	53	Female	Carcinoma	Invasive breast cancer	*LINC01197-NTRK3*	Likely fusion	Intrachromosomal rearrangement	6.1	MSS	Novel
28	57	Male	Carcinoma	Colorectal cancer	*ETV6-NTRK3*	Definite fusion	Interchromosomal rearrangement	180.3	MSI-H	Reported
29	59	Male	Carcinoma	Colorectal cancer	*TPM3-NTRK1*	Definite fusion	Intrachromosomal rearrangement	8.5	MSS	Reported
30	59	Female	Carcinoma	Ovarian serous cystadenocarcinoma	*ITGA4-NTRK3*	Definite fusion	Interchromosomal rearrangement	3.9	MSS	Novel
31	61	Female	Carcinoma	Colorectal cancer	*TPM3-NTRK1*	Definite fusion	Intrachromosomal rearrangement	8.5	MSS	Reported
32	61	Male	Carcinoma	Non-small cell lung cancer	*COL8A1-NTRK3*	Definite fusion	Interchromosomal rearrangement	10.8	MSS	Reported
33	63	Male	Carcinoma	Non-small cell lung cancer	*PRDX1-NTRK1*	Definite fusion	Intrachromosomal rearrangement	1.5	MSS	Novel
34	64	Male	Carcinoma	Hepatocellular carcinoma	*PEAR1-NTRK1*	Definite fusion	Intrachromosomal rearrangement	6.9	MSS	Reported
35	66	Male	Carcinoma	Extrahepatic cholangiocarcinoma	*MMP16-NTRK3*	Likely fusion	Interchromosomal rearrangement	6.1	MSS	Novel
36	67	Female	Soft tissue sarcoma	Uterine leiomyosarcoma	*MEF2A-NTRK3*	Definite fusion	Intrachromosomal rearrangement	3.8	MSS	Novel
37	69	Female	Carcinoma	Colorectal cancer	*TPM3-NTRK1*	Definite fusion	Intrachromosomal rearrangement	78.1	MSI-H	Reported
38	71	Female	Carcinoma	Colorectal cancer	*TPR-NTRK1*	Definite fusion	Intrachromosomal rearrangement	54.9	MSI-H	Reported
39	72	Male	Carcinoma	Colorectal cancer	*ETV6-NTRK3*	Definite fusion	Interchromosomal rearrangement	56.2	MSI-H	Reported
40	76	Male	Carcinoma	Colorectal cancer	*LMNA-NTRK1*	Definite fusion	Intrachromosomal rearrangement	6.2	MSS	Reported

Definite fusion: fusions with in-strand and in-frame state and the *NTRK* kinase domain.

Likely fusion: fusions with out-frame state in a known partner gene or in a hot exon and the *NTRK* kinase domain.

### *NTRK* fusions

*NTRK* fusion was predicted to be a functional TRK fusion protein, including “definite fusion” of in-strand and in-frame states with the *NTRK* kinase domain and “likely fusion” of out-frame states in a known partner gene or in a hot exon with the *NTRK* kinase domain.

**Fig. 1:**
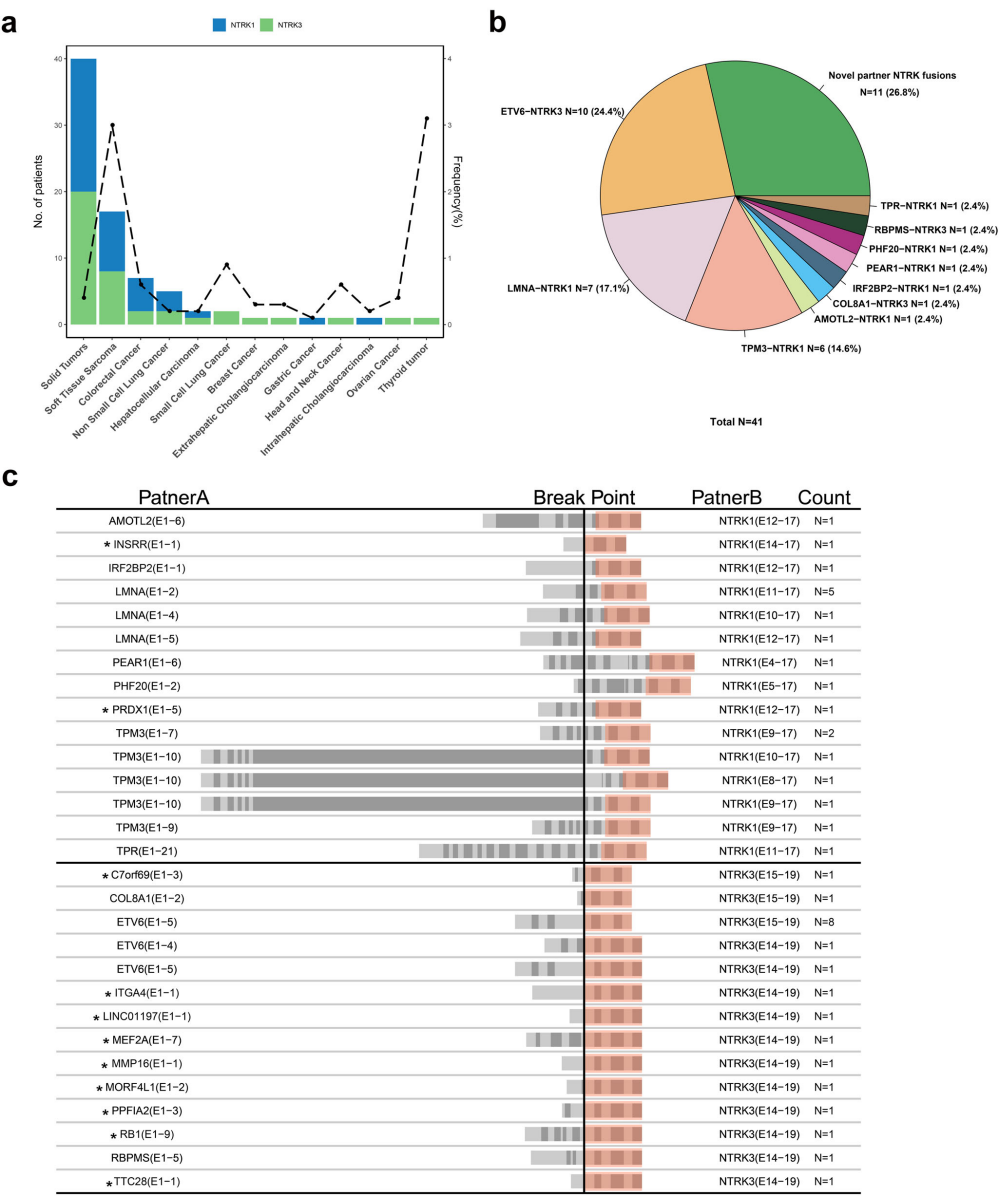
Overview of *NTRK* fusions in the Chinese population. **a** Distribution of *NTRK* fusion in various tumor types. The frequency of *NTRK* fusion in each cancer was denoted on the top of the bar. **b** Distribution of partner genes in *NTRK* fusions. **c** Structure of 29 *NTRK* fusion patterns and the positions of each breakpoint. The middle horizontal line distinguishes the fusions of *NTRK1* and *NTRK3*, the middle vertical line represents the breakpoint of gene fusions, and the orange color boxes represent the kinase domain of the *NTRK* genes. Asterisks indicate novel fusion patterns of *NTRK* genes.

In this cohort, a total of 41 *NTRK* fusions were detected in 40 patients, including 34 *NTRK* definite fusions and 7 *NTRK* likely fusions (Table 2). The incidence of *NTRK* fusions in tumor types was as follows: 3.0% in soft tissue sarcoma (17/571), 3.1% in thyroid tumor (1/32), 0.9% in small cell lung cancer (2/220), 0.6% in colorectal cancer (7/1225) and head and neck carcinoma (1/175), 0.4% in ovarian cancer (1/261), 0.3% in extrahepatic cholangiocarcinoma (1/351) and breast cancer (1/323), 0.2% in non-small cell lung cancer (5/2039), hepatocellular carcinoma (2/1133), and intrahepatic cholangiocarcinoma (1/555), and 0.1% in gastric cancer (1/866) (Fig. 1a), and detailed histologic diagnosis of these cases were listed in Table 2. There were 20 (48.8%) *NTRK1* and 21 (51.2%) *NTRK3* fusions in 7 and 12 tumor types, respectively (Fig. 1a). No *NTRK2* fusion was detected in this cohort.

The cohort included 55 fibrosarcoma patients, and 7 (12.7%) were *NTRK* fusion-positive. Given the age of these patients, *NTRK* fusion occurred significantly more frequently in infantile fibrosarcoma (age ≤ 18 years) (6/12) than in adult fibrosarcoma (age 18 years, 1/43) (*P* < 0.001).

Overall, 21 unique fusion partners were identified in our *NTRK* fusion-positive cohort (*N* = 40, Table 2). Several fusions are well known, including *ETV6-NTRK3* (*N* = 10), *LMNA-NTRK1* (*N* = 7), *TPM3-NTRK1* (*N* = 6), and *TPR-NTRK1* (*N* = 1); 11 were rare fusions (26.8%, 11/41) including 2 rare partners with *NTRK1* and 9 rare partners with *NTRK3* (Fig. 1b). *NTRK* fusion patterns and the exon composition of *NTRK* and their partners were identified in our study. Totally, *NTRK3* fusions had the most diverse partners (*N* = 12), followed by *NTRK1* fusions (*N* = 9). In our cohort, there was no fusion partner shared by *NTRK1* and *NTRK3* (Fig. 1c). All of the fusions preserved an intact tyrosine kinase domain, which could lead to constitutive activation of TRK receptors. *NTRK1* breakpoints were located on a broad range of introns, including introns 3, 4, 7, 8, 9, 10, 11, and 13, and exons 8, 10, and 11. By contrast, *NTRK3* breakpoints were limited to intron 13 and intron 14. This suggested that *NTRK1* and *NTRK 3* had distinctive fusion patterns in our cohort. Furthermore, intrachromosomal rearrangements comprised the most fusions of *NTRK1* (90%, 18/20), whereas interchromosomal rearrangements comprised the vast majority of *NTRK3* fusions (85.7%, 18/21) (Table 2). Most rare fusion partners and 70% (7/10) of known fusion partners were present only once. Additionally, breakpoints were distributed in different exons of the *NTRK* genes, especially for *NTRK1*, suggesting that *NTRK* fusions were heterogeneous in Chinese solid tumor patients.

### Co-occurring genomic alterations in *NTRK* fusion-positive patients

**Fig. 2:**
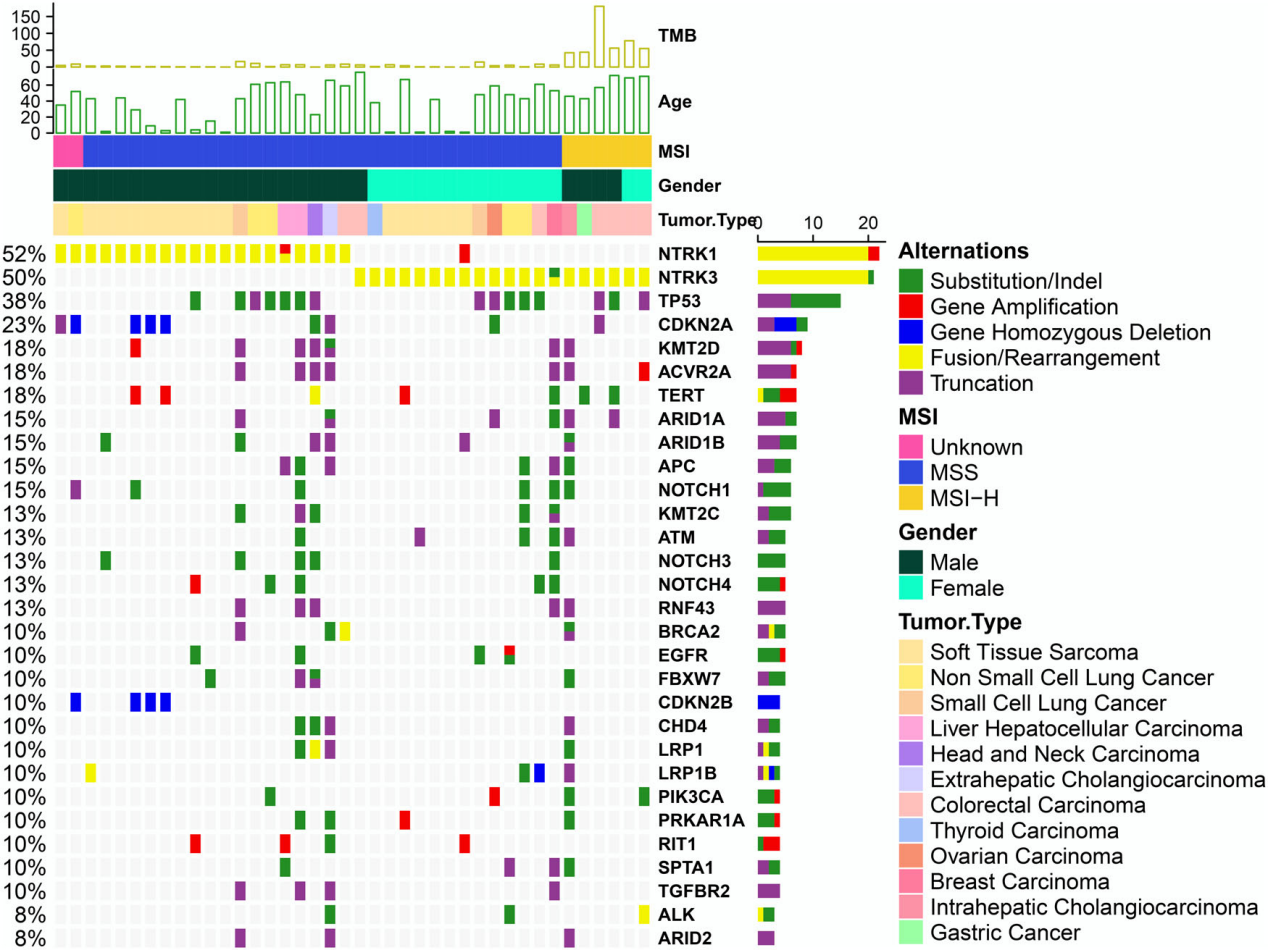
Comprehensive genomic profiling of 40 *NTRK* fusion-positive patients. The *X-axis* represents each patient and the *Y-axis* represents each mutated gene. The bar graph above shows the information of tumor mutational burden (TMB), age, MSI, and gender of each patient, and the bar graph on the right shows the mutation number of each sample. Green represents substitution/Indel mutations, red represents gene amplification mutations, blue represents gene homozygous deletion mutations, yellow represents fusion/rearrangement mutations, and purple represents truncation mutations.

One of the advantages of NGS technology is the simultaneous discovery of various genomic variants. The co-occurrence of multi-genomic alterations was identified in patients with *NTRK* fusions (Fig. 2).

Among 40 cases with *NTRK* fusions, 30% (12 of 40) harbored alterations that activated the downstream PI3K signaling pathway, including the alterations from *PIK3CA*, *PTEN*, *MORT*, *STK11*, *TSC1*, *RPTOR*, and *NF2*; 50% (20 of 40) harbored alterations within cell-cycle–associated genes, including *CDKN2A/B*, *RB1*, *CCND2*/*3*, *CDK4*, and *CCNE1*; 45% (18 of 40) harbored alterations within other tyrosine kinase receptor genes, including *EGFR*, *ERBB2/3*, *FGFR3/4*, *KDR*, *FLT4*, *PDGFRB*, *DDR2*, and *JAK3*; and 55% (22 of 40) harbored alterations within TP53-associated genes, including *TP53*, *ATM*, and *MDM2* (Supplementary Fig. 2).

The most enriched co-occurrence of mutated genes with *NTRK* fusions were *TP53* (38%, 15/40), *CDKN2A* (23%, 9/40), and *ACVR2A* (18%, 7/40). *CDKN2A/2B* homozygous deletion exclusively occurs in soft tissue sarcoma patients with *NTRK1* fusions. Alterations in *ATM* (4 vs. 1)*, LRP1B* (3 vs. 1), and *PTEN* (2 vs. 0) were more frequently observed in patients with *NTRK3* fusions than in those with *NTRK1* fusions. We also found a 42-year-old male patient (Patient 16) with stage I retroperitoneal liposarcoma who carried double *NTRK3* fusions, such as *MORF4L1*-*NTRK3* and *PPFIA2*-*NTRK3*, accompanied by the amplifications of *CDK4*, *IDH2*, *MDM2*, *TERT*, and *TNFSF11*.

Three *EGFR*-activated alterations coupled with *NTRK* fusions were detected in lung cancer patients, including two non-small cell lung cancer patients and one small cell lung cancer patient. These patients were previously treated with *EGFR* tyrosine kinase inhibitor (TKI). A 43-year-old female patient diagnosed with advanced non-small cell lung cancer with bone metastasis harbored the *EGFR* exon 19 deletion and received the treatment of erlotinib, and the mass of the right lung tumor was reduced after about 2 months of treatment. However, four more months later, an enlarged mass on the left lung tumor was observed, and the mutation of *EGFR* T790M was detected. After receiving the treatment of osimertinib for about 11 months, her disease progressed. Then the mutation of *EGFR* exon 19 deletion and a gene fusion of *TPM3*-*NTRK1* were detected but without the mutation of *EGFR* T790M. Besides, a single nucleotide variant (SNV) of *TP53* P278A was also detected.

### *NTRK* SNVs and amplifications

The frequency of *NTRK* amplification in our cohort with solid tumors was approximately 0.8% (*N* = 86). *NTRK* amplifications were detected in hepatocellular carcinoma (35/1133, 3.1%), soft tissue sarcoma (12/571, 2.1%), intrahepatic cholangiocarcinoma (8/555, 1.4%), gastric cancer (4/866, 0.5%), and non-small cell lung cancer (4/2039, 0.2%) (Supplementary Fig. 3a). The most frequent *NTRK* amplification occurred in *NTRK1* (92%, 79/86), followed by *NTRK3* (7%, 6/86), and *NTRK2* (1%, 1/86).

The frequency of *NTRK* SNVs was higher than that of *NTRK* fusions and *NTRK* amplifications. The frequency of *NTRK* SNVs in our cohort with solid tumors was approximately 3.0% (*N* = 302). The *NTRK* SNVs were frequently detected in colorectal cancer (68/1225, 5.6%), non-small cell lung cancer (62/2039, 3.0%), gastric cancer (43/886, 5.0%), hepatocellular carcinoma (24/1133, 2.1%), and small cell lung cancer (13/220, 5.9%) (Supplementary Fig. 3b). The most frequent *NTRK* SNV occurred in *NTRK3* (54.3%, 164/302), followed by *NTRK2* (24.5%, 74/302), and *NTRK1* (21.1%, 64/302). Variants in the solvent front, the gatekeeper, or the xDFG motif of the kinase domain have been reported as acquired resistant variants to TRK inhibitors such as *NTRK1* G595R/F589L/G667C/S, *NTRK2* G639R/F633L/G709C, and *NTRK3* G696A/G623R/F617L^13,14^. These variants were not observed in any of the 10,194 combined adult and pediatric tumors.

We also found that *NTRK* amplifications and *NTRK* SNVs rarely occurred with *NTRK* fusions, which were 5.0% (2/40) and 2.5% (1/40), respectively. Furthermore, tumors with *NTRK* SNVs had higher TMB than those with *NTRK* amplifications, *NTRK* fusions, or those without *NTRK* alteration (*P* < 0.001, respectively) (Supplementary Fig. 4).

### Comparison of immunohistochemistry, DNA-, and RNA-based NGS assays

The *NTRK* fusions can be detected by multiple platforms, such as FISH, RT-PCR, or NGS, and the overexpression of TRK proteins caused by fusion events can be confirmed by IHC. In our study, 13 *NTRK* fusion-positive samples detected by DNA-based NGS assay also underwent RNA-based NGS assay, and TRK expression in 9 samples was further confirmed by IHC detection. Results showed that for 12 of 13 patients, *NTRK* fusions were also identified by RNA-seq (Table 3). For one remaining patient (Patient 11) with a likely fusion of *C7orf69*-*NTRK3*, RNA-seq did not confirm the presence of the fusion transcript. However, due to insufficient samples, we failed to conduct further validation by NGS. For an infantile fibrosarcoma patient (patient 2, Table 3) and a colorectal carcinoma patient (patient 39, Table 3), the fusions of *ETV6-NTRK3* were detected by both DNA- and RNA-based NGS assays, but the pan-TRK IHC was negative. Consistent with the results of DNA- and RNA-based NGS detection, the expression of NTRK was confirmed by IHC in seven samples (Table 3). Together, our results indicate that NGS detection based on the combination of DNA and RNA can effectively meet clinical *NTRK* fusion detection.

**Table 3:** The comparison of immunohistochemistry, DNA-, and RNA-based NGS assays.

Patient	Tumor_type	Fusion at DNA level	NGS RNA-seq	NGS DNA-seq	IHC
2	Infantile fibrosarcoma	*ETV6* exon1-5-*NTRK3* exon15–19	Positive	Positive	Negative
5	Cellular plexiform schwannoma	*TPM3* exon1-10-*NTRK1* exon9–17	Positive	Positive	Positive
7	Rhabdomyosarcoma	*LMNA* exon1-5-*NTRK1* exon8–17	Positive	Positive	Positive
8	Infantile fibrosarcoma	*TPM3* exon1-9-*NTRK1* exon9–17	Positive	Positive	/^a^
9	Soft tissue sarcoma of the abdomen	*LMNA* exon1-2-*NTRK1* exon10–17	Positive	Positive	/
10	Soft tissue sarcoma of the abdomen	*LMNA* exon1-2-*NTRK1* exon11–17	Positive	Positive	Positive
11	Mucoepidermoid carcinoma of trachea	*C7orf69* exon1-3-*NTRK3* exon15–19	Negative	Positive	/
12	Spindle cell sarcoma of the prostate	*RBPMS* exon1-5-*NTRK3* exon14–19	Positive	Positive	/
13	Prostatic stromal tumor	*IRF2BP2* exon1-*NTRK1* exon17	Positive	Positive	Positive
15	Spindle cell sarcoma of the thigh	*LMNA* exon1-2-*NTRK1* exon11–17	Positive	Positive	Positive
17	Spindle cell tumors of the sacrum	*LMNA* exon1-2-*NTRK1* exon11–17	Positive	Positive	Positive
29	Colorectal_cancer	*TPM3* exon1-7-*NTRK1* exon9–17	Positive	Positive	Positive
38	Colorectal_cancer	*TPR* exon1-21-*NTRK1* exon11–17	Positive	Positive	Negative

^a^Not evaluated.

### Clinical response for *NTRK* fusion-positive patients

**Fig. 3:**
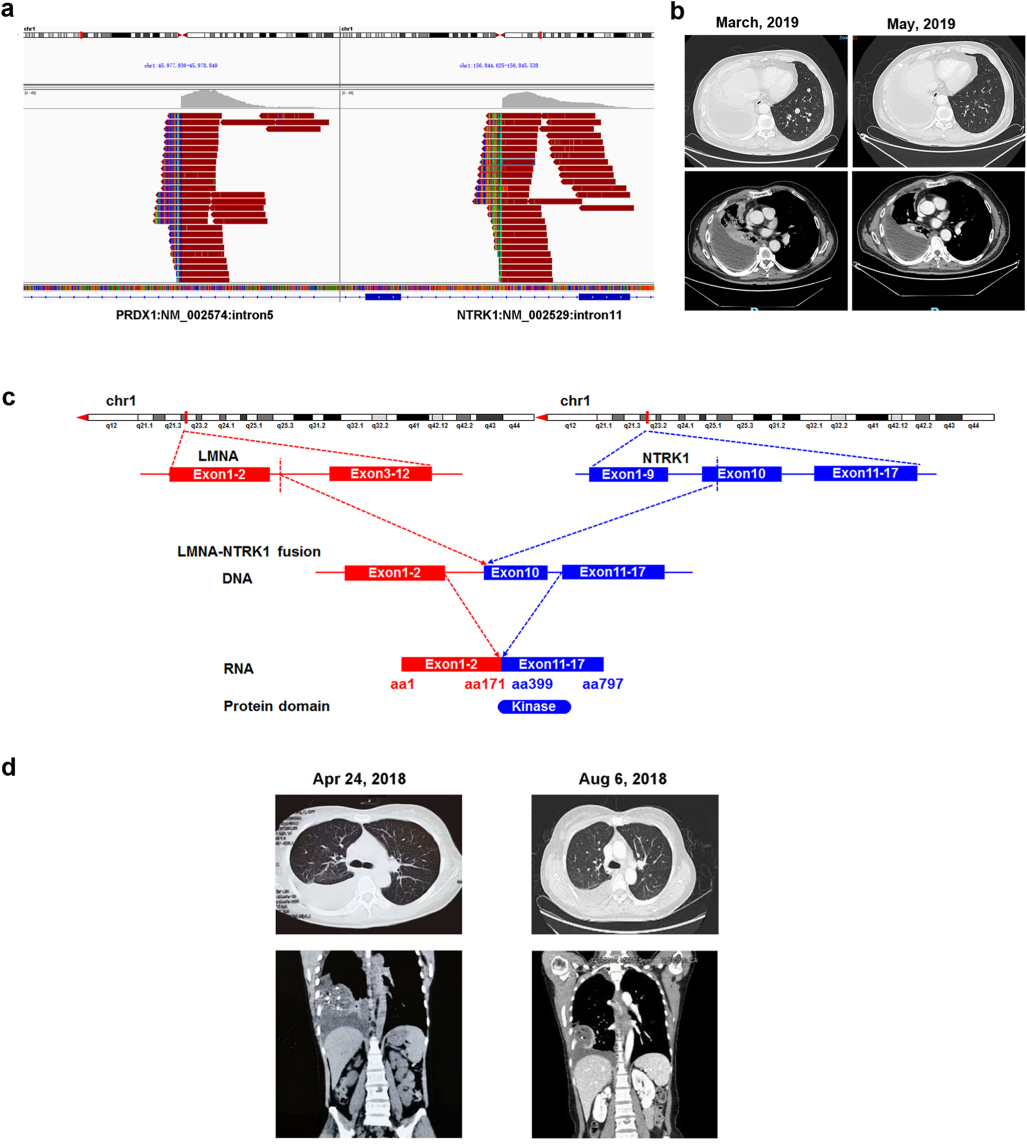
The clinical response of patients with *NTRK* fusion to larotrectinib. **a** The *PRDX1* exon 5-*NTRK1* exon 12 fusion in Integrative genomics viewer (IGV) from the patient (Patient 33) with lung adenocarcinoma. IGV screenshot showed the breakpoints on the intron 5 of *PRDX1* gene (left) and on the intron 11 of *NTRK1* gene (right) detected by capture-based next-generation sequencing. **b** CT scans of Patient 33 before and after 2 months of larotrectinib treatment. **c** The illustration of *LMNA-NTRK1* fusion at DNA and RNA levels in Patient 15. **d** Radiological comparison of abdominopelvic CT scans before and after 2 months of larotrectinib treatment by coronal and transverse planes of Patient 15.

In our study, two patients underwent treatment with TKIs, such as larotrecitnib. The first patient (Patient 33) is a 63-year-old Chinese male who was diagnosed with lung adenocarcinoma accompanied by mediastinal lymph node metastasis and double lung metastasis with a rare *PRDX1*-*NTRK1* fusion (Fig. 3a) and an *MTOR* E2419D mutation. A pan-TRK IHC assay also indicated that this patient was TRK-positive. Subsequently, he was treated with crizotinib at a dose of 85 mg twice a day. One month later, this patient achieved a partial response with a reduction in lung lesions and the disappearance of pleural effusion. After 4 months of crizotinib treatment, he received the combination therapy of pemetrexed and cisplatin for 5 months. Subsequently, he was successfully recruited for an ongoing clinical trial of the selective TRK inhibitor larotrectinib, and the treatment started in March 2019. The patient experienced partial remission after 2 months (Fig. 3b) and had a sustained durable response to larotrectinib until April 2021.

The second patient (Patient 15) is a 42-year-old Chinese female who was diagnosed with advanced spindle cell fibrosarcoma of the thigh in 2009. Over the subsequent 9 years, she underwent multiple lines of treatment without achieving effective control of the disease. In March 2018, an *LMNA-NTRK1* fusion involving exons 1 and 2 of *LMNA* and exons 10–17 of the *NTRK1* gene was identified by NGS (Supplementary Fig. 5). The *LMNA*-*NTRK1* fusion detected by DNA-seq and RNA-seq is illustrated in Fig. 3c. She was recruited for an ongoing clinical trial of the selective TRK inhibitor larotrectinib, and the treatment was started in June 2018. After 2 months of treatment, a CT scan revealed stable disease according to RECIST1.1 (Fig. 3d). To our knowledge, this is a rare report on the clinical response to larotrectinib in a Chinese patient with spindle cell fibrosarcoma harboring an *LMNA*-*NTRK1* fusion.

## Discussion

Gene fusions involving *ALK* and *ROS1* have been increasingly recognized as clinically actionable alterations. *NTRK* fusions have been reported to promote tumorigenesis by constitutively activating downstream cell growth and proliferative pathways, resulting in pathways addiction and making them attractive targets of cancer therapy^15^. In addition, the presence of *NTRK* fusions in certain cancers, such as non-small cell lung cancer, is associated with poor survival^16^. Although *NTRK* fusions are infrequent in solid tumors (typically seen in <1% of patients), both the present data and previous reports demonstrate that *NTRK* fusions are enriched in certain histologic subsets, including pediatric fibrosarcomas, secretory carcinoma of the breast, and mammary analog secretory carcinoma^12,17^.

In the present study, 0.4% (40/10,194) Chinese solid tumor patients harbored *NTRK* fusions, whereas, in a report on a Western cohort, 0.27% of 11,502 solid tumor patients were found to have *NTRK* fusions^18^. Of note, the frequency of *NTRK1* fusion in non-small cell lung cancer was higher than that from another report in the Chinese population (0.17% vs. 0.073%)^19^. This is a large-scale study on the distribution of *NTRK* fusions in Chinese patients with a variety of solid tumors. Consistent with previous studies^20,21^, our findings also showed a relatively high frequency of *NTRK* fusions in patients with fibrosarcoma and thyroid tumor. Several *NTRK* fusions identified in our study have been previously reported as activating and oncogenic fusions involved in cancer cell growth, proliferation, and poor survival^4,16,22^. Protein domain analyses indicate that these fusions, as well as the 11 rare fusions, contain the TRK tyrosine kinase domain regardless of the 5’ fusion partner.

The fusion of *NTRK3* is rare in tumors, and the fusion of *NTRK3* with two partner genes in one patient is even rarer. We identified two *NTRK3* fusions in a male patient, namely *MORF4L1-NTRK3* and *PPFIA2-NTRK3*. The breakpoint of *NTRK3* both occurred in intron 13, while the partner genes were located on chromosome 15 (*MORF4L1*) and chromosome 12 (*PPFIA2*), respectively. According to the results of genome alignment, the fusions of *MORF4L1-NTRK3* and *PPFIA2-NTRK3* can be supported by 255 and 198 pairs of reads, respectively. However, the lack of sufficient samples for further validation is a limitation of this study. Based on the NGS-based genomic detections, we explored the mutational characteristics of 40 patients with *NTRK* fusions. Our results showed that *NTRK* fusions co-occur with *TP53*-associated genes and cell cycle-associated genes. Rosen et al. reported that *NTRK* fusions were rarely co-mutated with other canonical oncogenes, including *EGFR*^23^. Previous reports showed that *NTRK* fusion might be the acquired resistant variant for the *EGFR* TKI in lung cancer patients^24^. In this study, *EGFR* variants were detected in 10% of patients with *NTRK* fusions. For one patient (patient 18), the *TPM3-NTRK1* fusion was identified after the disease progressed on osimertinib. In this patient, the *TPM3*-*NTRK1* fusion may be a potential mechanism of acquired osimertinib resistance^17,25^. TRK inhibitors have attracted considerable attention in the past few years due to the dramatic, long-lasting response observed in patients with *NTRK* fusion who received therapy in early clinical trials^22,26^. Though multitargeted tyrosine kinase inhibitors such as crizotinib demonstrated efficacy in patients carrying the *NTRK1* fusion^22,27,28^, selective TRK inhibitors revealed superior efficacy. Larotrectinib achieves a 78% objective response rate in tumors harboring *NTRK* fusion^4,6,29,30^, and it was approved by the FDA for the treatment of metastatic solid tumors carrying *NTRK* fusion, regardless of the underlying tumor histology^12^. Since novel selective inhibitors of constitutively active rearranged proteins have been developed, the ability to detect *NTRK* fusions will have a significant impact on clinical practice.

Similar to the previous study^17^, we also identified an association between *NTRK* fusion and MSI-H. The development of colorectal cancer is often accompanied by the occurrence of MSI-H or chromosomal instability^25^. This explains the high proportion of colorectal cancer in *NTRK* fusion-positive patients with MSI-H. MSI-H and *NTRK* fusion positivity are both biomarkers of pan-cancer. MSI-H is associated with high immune scores and a higher response to immune checkpoint inhibitors^31,32^, while NTRK has dual functions of promoting nervous system development and carcinogenesis^33,34^. TRK inhibitors such as larotrectinib have shown good efficacy in patients with *NTRK* fusions^22,26^. However, in the presence of two biomarkers simultaneously, the efficacy of monotherapy and combination therapy is intriguing, and further clinical research is needed to confirm.

NGS-based DNA detection can maximize the detection of genomic target site variations, while NGS-based RNA detection can identify target genes at the transcriptome level, which is conducive to increasing the fusion detection rate and discovering unknown fusions^35^. FISH is well established as the diagnostic gold standard for fusion gene detection^36^. Ben et al. also showed that the combination of DNA-seq and RNA-seq could effectively enhance the detection of gene fusions^37^. In our study, 12 of 13 *NTRK* fusion-positive cases detected by DNA-based NGS were also detected by RNA-based NGS assay. The failure of *NTRK* fusion detection in one sample at the RNA level was probably caused by the low level of transcription or degradation due to sample handling or by nonsense-mediated decay. This sample was also negative for IHC. One common fusion, *ETV6*-*NTRK3* (Patient 2, Table 3), was detected by both NGS-based RNA-seq and DNA-seq, but the protein was not detected by pan-TRK IHC. This might have been caused by protein degradation, which showed the limitations of this technology. Together, NGS-based DNA-seq and RNA-seq can confirm each other, and simultaneously detecting both is the better strategy for gene fusion detection. Two of our patients with *NTRK* fusions at the DNA level and known treatment histories received crizotinib or larotrectinib treatment and showed good clinical responses. This implies the advantage of NGS for *NTRK* fusion detection: It allows numerous genes to be analyzed in a much less labor-intensive manner than that with Sanger sequencing and detects more variant types, such as gene-activating SNVs, in-frame indel, and amplification or rearrangements, which contributes to a more comprehensive clinical landscape and more precision clinical practice.

In conclusion, we identified nearly 0.4% of *NTRK* fusion events in a large Chinese cohort, described the mutational characterization of *NTRK* fusion-positive patients, and reported the real cases of patients with *NTRK* fusion who benefit from larotrectinib. Our results showed that NGS detection, including DNA-based and the combination of DNA-based and RNA-based, can effectively assist clinical *NTRK* fusion detection and is of great significance for the identification of novel *NTRK* fusions. This study profiled the prevalence and molecular distribution of *NTRK* fusions in Chinese solid tumor patients, which supported the application of NGS to clinical oncology practice and guided the use of TRK inhibitors to help patients with such rare genomic alterations improve their clinical performance.

## Methods

### Patients

This study was conducted according to the Declaration of Helsinki and approved by the Institutional Review Board of the Shandong Provincial Hospital and the Shanghai Ethics Committee for Clinical Research. All patients provided written informed consent. From 2017 to 2018, a total of 10,194 pathologically diagnosed solid tumor samples, either resected or biopsied, were collected from patients across all of China, including 4222 (41.4%) from East China, 2983 (29.3%) from South China, 966 (9.5%) from Southwest China, 953 (9.3%) from North China, 505 (5.0%) from Central China, 333 (3.3%) from Northwest China, and 232 (2.3%) from Northeast China^38^. Formalin-fixed paraffin-embedded (FFPE) tumor tissues and matched blood samples were collected to detect genomic alterations.

### Next-generation sequencing

All samples were subjected to sequencing at OrigiMed (OrigiMed, Inc., Shanghai), a College of American Pathologists (CAP) and Clinical Laboratory Improvement Amendments (CLIA) certified laboratory (Certificate ID: 99D2159871). Genomic DNA was extracted from 10,194 samples using a DNA Extraction Kit (QIAamp DNA FFPE Tissue Kit) according to the manufacturer’s protocols. And typically, 50–250 ng of double-stranded DNA was fragmented to about 250 bp by sonication. Subsequent library construction using the KAPA Hyper Prep Kit (KAPA Biosystems) for end repair, dA addition, and adapter ligation was performed, followed by PCR amplification and quantified by Qubit assessment.

RNA was successfully extracted from unstained FFPE sections of 356 cases (miRNeasy FFPE Kit, Qiagen) according to the manufacturer’s protocol. A cDNA primer mixture of random hexamer and oligo dT (Thermo Fisher Scientific) was annealed to the template RNA at 70 °C for 5 min. First strand synthesis was performed using M-MLV RT RNase(H-) (Promega) and followed by second strand synthesis (NEB). The cDNA was cleaned up using 1.8× Agencourt RNA Clean XP Beads (Beckman). The entire cDNA product was sheared by sonication (E220, Covaris) to the fragment of about 200 bp before library construction. Adapters were ligated to the libraries (KAPA Hyper Prep Kit, Roche) and quantified by the Qubit dsDNA HS Assay Kit (Thermo Fisher Scientific).

A custom hybridization panel with probes against all exons and selected introns (such as *NTRK1* introns 3–23, *NTRK2* introns 11, 12, and 15, and *NTRK3* introns 10 and 12–15) of *NTRK1*, *NTRK2*, and *NTRK3* that applied to DNA-seq and a custom hybridization panel with probes against all exons of *NTRK1*, *NTRK2*, and *NTRK3* that applied to RNA-seq were used to capture the targeted sequences followed the protocol of “Hybridization capture of DNA libraries using xGen® Lockdown® Probes and Reagents” (Integrated DNA Technologies). Post-capture libraries were mixed together, denatured and diluted to 1.5–1.8 pM, and subsequently sequenced on Illumina NextSeq 500. Tumor samples were sequenced to a median unique coverage of 1202× and matched normal blood samples were sequenced to a mean unique coverage of 300×.

### Bioinformatics pipeline for genomic alteration

Resultant sequences were mapped to the hg19 reference genome with BWA (version 0.7.12). SNVs were identified using MuTect (v1.7); short Insertions/deletions (Indels) were identified using PINDEL (V0.2.5); copy number variations (CNVs) were identified using EXCAVATOR (v2.2, http://sourceforge.net/projects/excavatortool/); TMB was calculated by counting the number of coding SNVs and indels per megabase of the sequence examined; the state of microsatellite stability was determined by candidate MSI markers including 572 identified microsatellite loci, and MSI-H is defined as more than 15% of selected microsatellite loci showing unstable in tumors compared to matched peripheral blood^39,40^. The in-house developed algorithm was used for DNA fusion detection and is detailed as follows: aligned reads with an abnormal insert size of over 2000 bp or matched to two different chromosomes were collected and used as discordant reads, i.e., paired-end reads that could not be closely mapped to a genome reference, with each read of paired-reads aligned to the same chromosomes or different chromosomes. Next, the discordant reads with a distance less than 500 bp formed clusters that were further assembled by Fermi-lite to identify potential rearrangement breakpoints^40,41^. The breakpoints were double-confirmed by BLAT, and the resulting chimeric gene candidates were annotated. At least five unique supporting read pairs were necessary for a genomic alteration. For RNA-Seq data, the STAR (v 2.5.3) algorithm was used to locate RNA-Seq readings, and STAR fusion (version 0.8) was used for fusion detection^42^. Gene fusion or rearrangements were finally assessed by Integrative Genomics Viewer (IGV).

### Pan-TRK immunohistochemistry

Briefly, slides were baked at 60 °C, deparaffinized in xylenes, and rehydrated with graded ethanol to distilled water. Antigen retrieval was performed using a Dako EnVision FLEX Target Retrieval Solution, high pH, in a steamer. Cooled slides were stained using an automated IHC staining platform. IHC staining for TRK A, B, and C expression was performed with a pan-TRK monoclonal antibody (mAb) clone EPR17341 (Abcam, Cambridge, MA)^9,10^. This antibody is reactive to a homologous region of TRKA, TRKB, and TRKC near the C terminus. All other staining was performed primarily with the Dako series reagents (K8002). EnVision FLEX+ wash buffer was used between incubation steps, and slides were counterstained with hematoxylin. Slides were then rinsed in distilled water and subjected to an ethanol dehydration series and xylene changes before coverslipping. Brain tissue was used as a positive control, and colorectal epithelium was used as a negative external control. Specimens were scored positive by pathologists if the specimens exhibited any staining intensity in >5% of tumor cells. Specimens without any visible or faint staining in tumor cells were scored negative.

### Statistical analysis

Statistical analysis was conducted using the R Statistical Software package (version 3.4.3, R Foundation for Statistical Computing, Vienna, Austria). Categorical variables are presented as numbers and percentages; medians and percentiles are reported for continuous variables. In multiple-group comparisons, Kruskal–Wallis rank-sum tests, Chi-square tests, or Fisher’s exact tests, with Bonferroni post-hoc comparisons, were used. The threshold for statistical significance was set at *P* < 0.05. The Circos plot was generated using the online Circos Table Viewer (http://mkweb.bcgsc.ca/tableviewer).

### Reporting summary

Further information on research design is available in the Nature Research Reporting Summary linked to this article.

### Supplementary information

Supplementary information

REPORTING SUMMARY

## Data Availability

All genomic data used in this study are available online (https://www.cbioportal.org/study/summary?id=pan_origimed _2020). All processed data generated for this study are available from the authors upon request.

## Code availability

All codes used in this manuscript are publicly available [http://ftp.origimed.com/gravityproject].
