# Supplementary material for: Prevalence and clinico-genomic characteristics of patients with TRK fusion cancer in China
Source: NPJ Precis Oncol. 2023 Aug 11;7:75. doi: 10.1038/s41698-023-00427-3 (PMC10421940; doi:10.1038/s41698-023-00427-3)

Supplementary data

Supplementary Figure 1. Distribution of the 10,194 solid tumor patients over 25 cancer types.

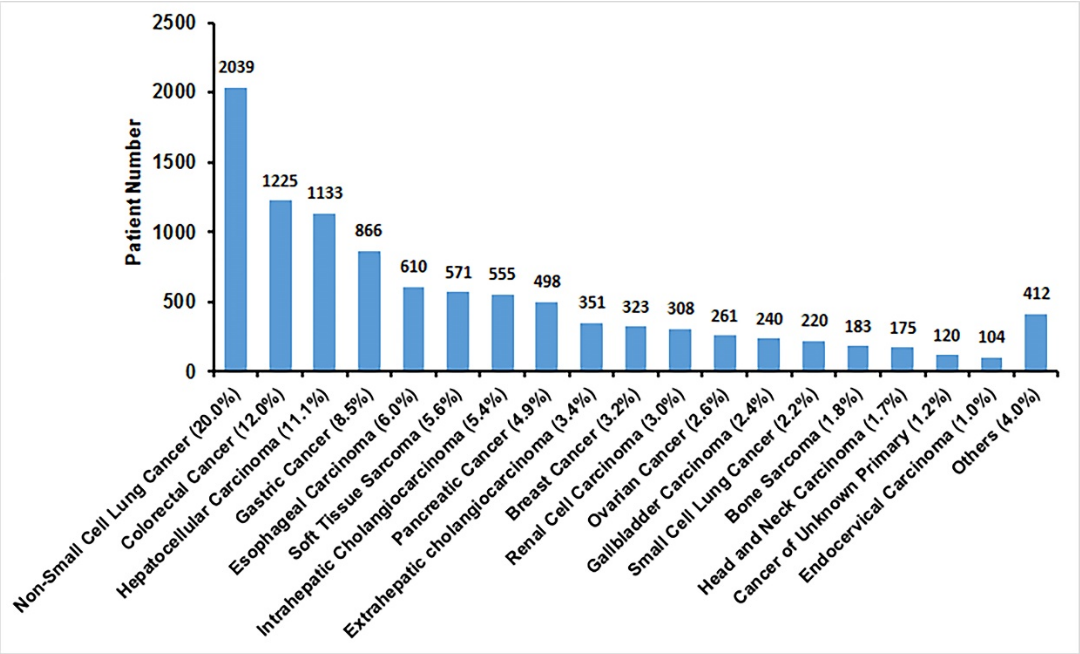

Supplementary Figure 2. Co-alterations associated with neurotrophic-tropomyosin receptor tyrosine kinase (*NTRK*) fusions in this study. The figure shows co-alterations including TP53-associated genes, cell cycle–associated genes, tyrosine kinase families, and phosphoinositide-3-kinase (PI3K) signaling alterations.

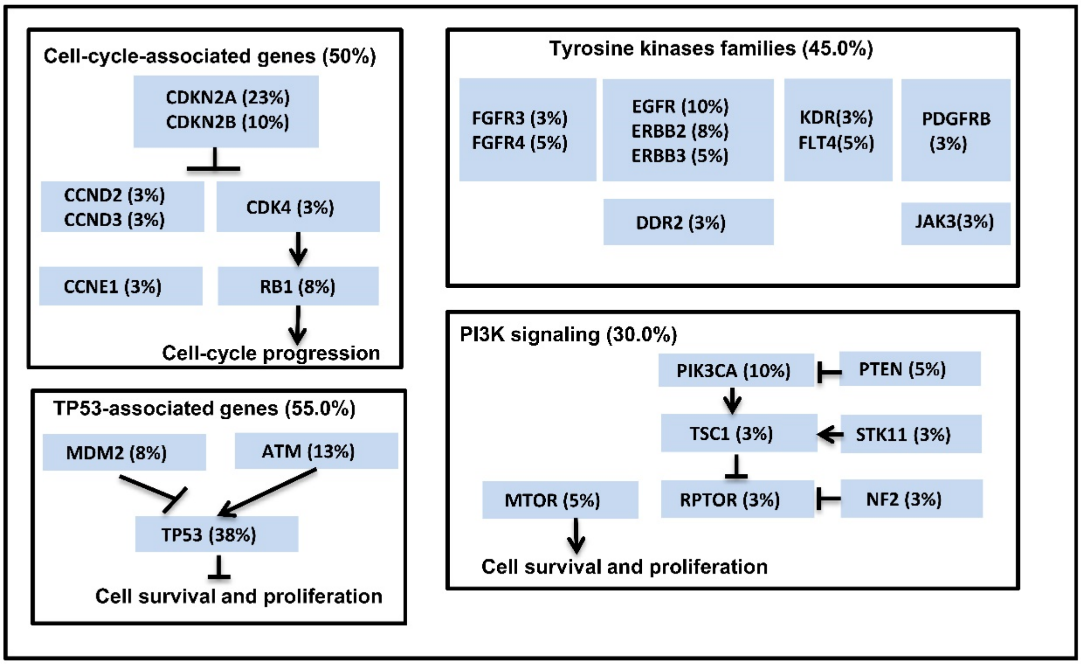

Supplementary Figure 3. The distribution of *NTRK* alterations in various tumor types. a Distribution of *NTRK* amplification. The prevalence of *NTRK* amplification in each cancer was denoted on the top of the bar. **b** Distribution of *NTRK* single nucleotide variants. The prevalence of *NTRK* single nucleotide variants in each cancer was denoted on the top of the bar.

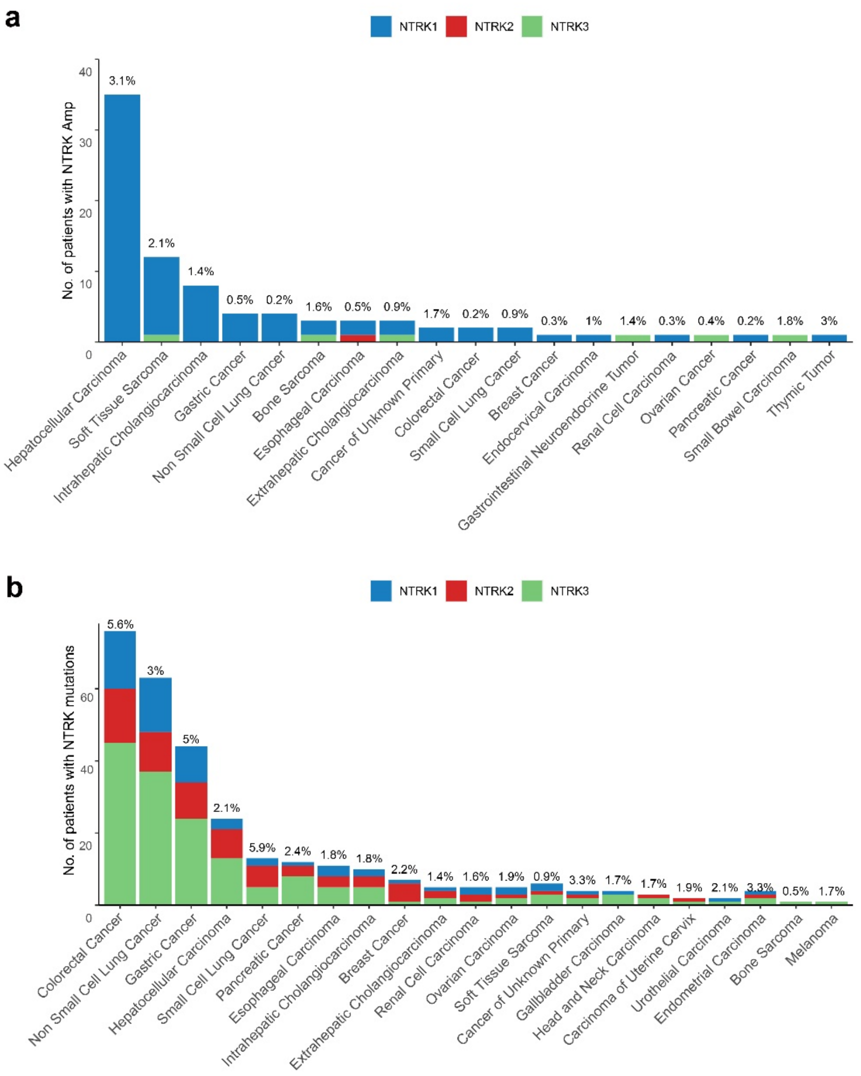

Supplementary Figure 4. Comparison of TMB among groups classified by *NTRK* alteration subtypes. A box-and-whisker plot is used to represent the data. Box plot represents first (lower bound) quartile, median, and third (upper bound) quartile. Whiskers, representing 1.5 times the interquartile range, were used to visualize data for these comparisons. Kruskal-Wallis rank sum tests were used for comparisons of TMB across four groups. Dots represent individual tumors.

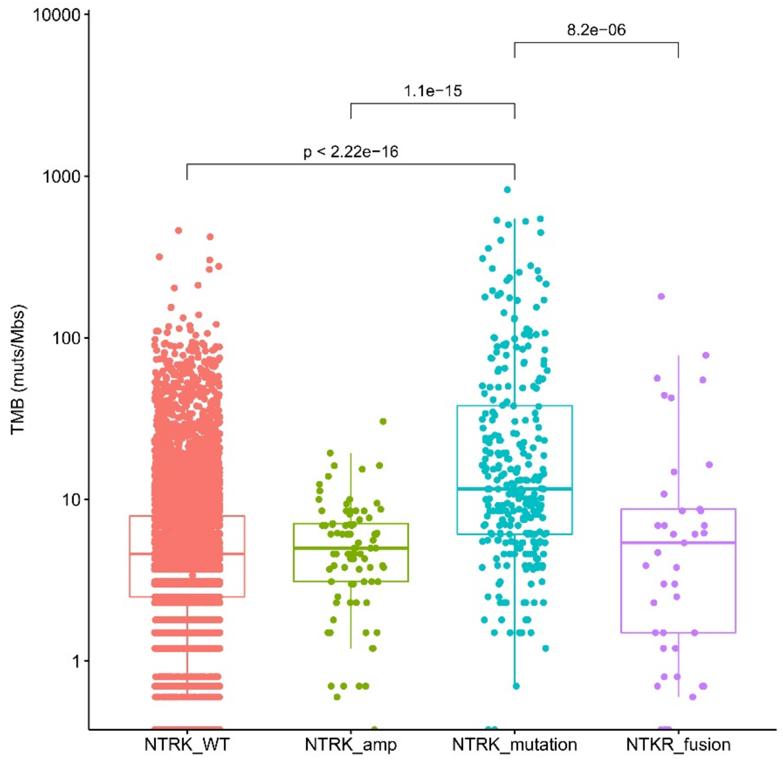

Supplementary Figure 5. The assessment of *NTRK* fusion detected by DNA- and RNA-based NGS with the Integrative Genomics Viewer. a *LMNA* exon2-*NTRK1* exon11 fusion at the DNA level (DNA supporting unique paired reads) detected in Patient 15. **b** *LMNA* exon 2-*NTRK1* exon10 fusion at the RNA level (RNA supporting unique paired reads) detected in Patient 15.

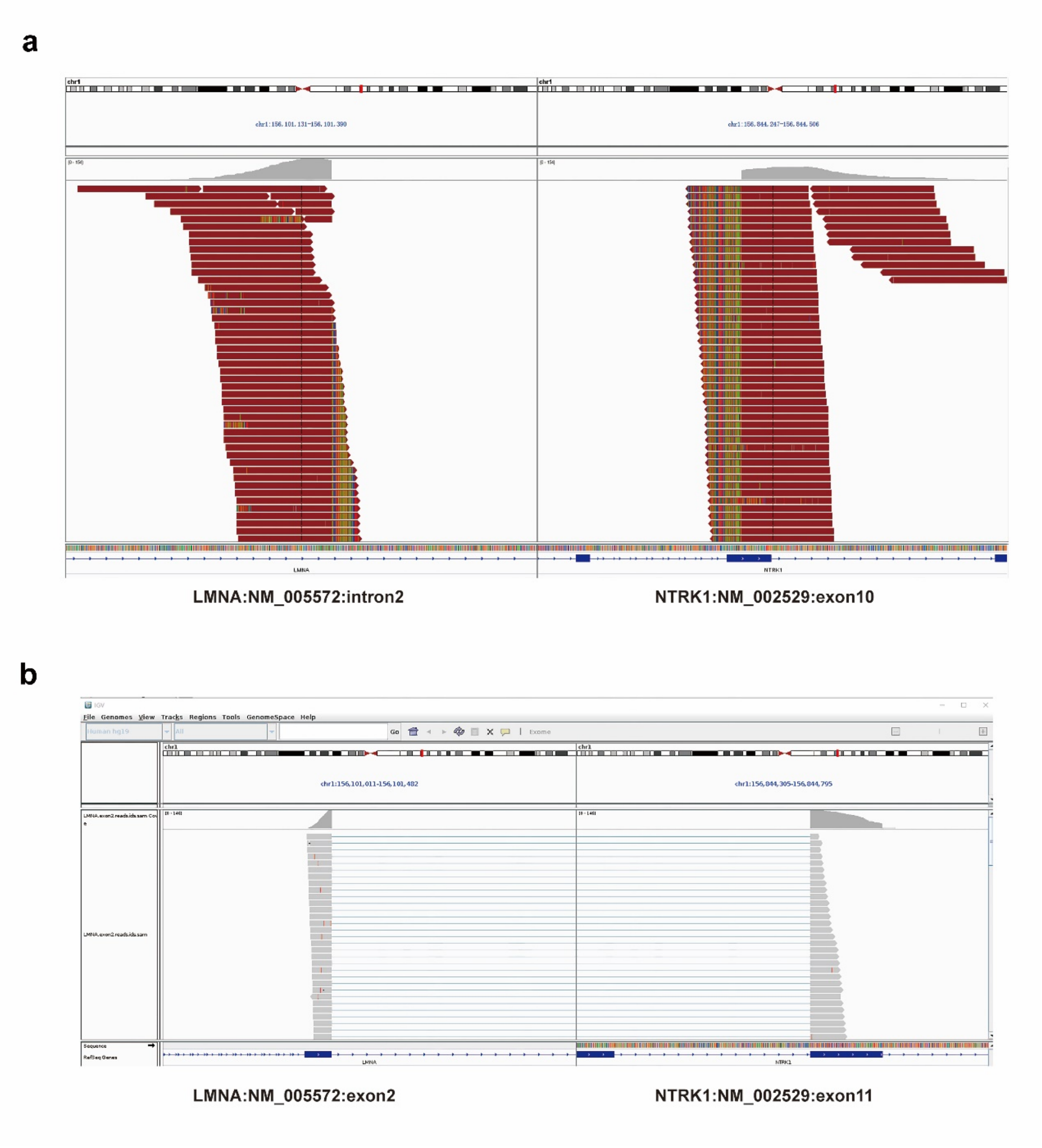

Supplement: Supplementary file 1 — Supplementary information [file 41698_2023_427_MOESM1_ESM.pdf]
