# Supplementary material for: Prevalence and clinico-genomic characteristics of patients with TRK fusion cancer in China
Source: NPJ Precis Oncol. 2023 Aug 11;7:75. doi: 10.1038/s41698-023-00427-3 (PMC10421940; doi:10.1038/s41698-023-00427-3)
Supplement: Supplementary file 2 — REPORTING SUMMARY [file 41698_2023_427_MOESM2_ESM.pdf]

nature portfolio

Corresponding author(s): Kai Wang

Last updated by author(s): Jul 5, 2023

Reporting Summary

Nature Portfolio wishes to improve the reproducibility of the work that we publish. This form provides structure for consistency and transparency in reporting. For further information on Nature Portfolio policies, see our Editorial Policies and the Editorial Policy Checklist.

Statistics

For all statistical analyses, confirm that the following items are present in the figure legend, table legend, main text, or Methods section.

n/a	Confirmed	
☐	☒	The exact sample size (*n*) for each experimental group/condition, given as a discrete number and unit of measurement
☐	☒	A statement on whether measurements were taken from distinct samples or whether the same sample was measured repeatedly
☐	☒	The statistical test(s) used AND whether they are one- or two-sided<br>*Only common tests should be described solely by name; describe more complex techniques in the Methods section.*
☒	☐	A description of all covariates tested
☒	☐	A description of any assumptions or corrections, such as tests of normality and adjustment for multiple comparisons
☒	☐	A full description of the statistical parameters including central tendency (e.g. means) or other basic estimates (e.g. regression coefficient) AND variation (e.g. standard deviation) or associated estimates of uncertainty (e.g. confidence intervals)
☒	☐	For null hypothesis testing, the test statistic (e.g. *F*, *t*, *r*) with confidence intervals, effect sizes, degrees of freedom and *P* value noted<br>*Give P values as exact values whenever suitable.*
☒	☐	For Bayesian analysis, information on the choice of priors and Markov chain Monte Carlo settings
☒	☐	For hierarchical and complex designs, identification of the appropriate level for tests and full reporting of outcomes
☒	☐	Estimates of effect sizes (e.g. Cohen's *d*, Pearson's *r*), indicating how they were calculated

Our web collection on statistics for biologists contains articles on many of the points above.

Software and code

Policy information about availability of computer code

Data collection	No software was used.
Data analysis	Statistical analysis was conducted using the R Statistical Software package (version 3.4.3, R Foundation for Statistical Computing, Vienna, Austria). Categorical variables are presented as numbers and percentages; medians and percentiles are reported for continuous variables. In multiple-group comparisons, Kruskal-Wallis rank-sum tests, Chi-square tests, or Fisher's exact tests, with Bonferroni post-hoc comparisons, were used. The threshold for statistical significance was set at p < 0.05. The Circos plot was generated using the online Circos Table Viewer (http://mkweb.bcgsc.ca/tableviewer).

For manuscripts utilizing custom algorithms or software that are central to the research but not yet described in published literature, software must be made available to editors and reviewers. We strongly encourage code deposition in a community repository (e.g. GitHub). See the Nature Portfolio guidelines for submitting code & software for further information.

Data

Policy information about availability of data

All manuscripts must include a data availability statement. This statement should provide the following information, where applicable:

- Accession codes, unique identifiers, or web links for publicly available datasets
- A description of any restrictions on data availability
- For clinical datasets or third party data, please ensure that the statement adheres to our policy

All genomic data used in this study are available online (https://www.cbioportal.org/study/summary?id=pan_origimed_2020). All processed data generated for this study are available from the authors upon request.

Research involving human participants, their data, or biological material

Policy information about studies with human participants or human data. See also policy information about sex, gender (identity/presentation), and sexual orientation and race, ethnicity and racism.

Reporting on sex and gender	In the cohort, 60.4% were male and 39.6% were female. This study does not involve sex- and gender- based analysis.
Reporting on race, ethnicity, or other socially relevant groupings	This study does not involve analysis based on race, ethnicity, and other socially relevant groupings.
Population characteristics	The cohort (Figure S1, Table 1) included non-small cell lung cancer (N=2039, 20.0%), colorectal cancer (N=1225, 12.0%), hepatocellular carcinoma (N=1133, 11.1%), gastric cancer (N=866, 8.5%), esophageal carcinoma (N=610, 6.0%), soft tissue sarcoma (N=571, 5.6%), intrahepatic cholangiocarcinoma (N=555, 5.4%), pancreatic cancer (N=498, 4.9%), extrahepatic cholangiocarcinoma (N=351, 3.4%), breast cancer (N=323, 3.2%), renal cell carcinoma (N=308, 3.0%), ovarian cancer (N=261, 2.6%), gallbladder carcinoma (N=240, 2.4%), small cell lung cancer (N=220, 2.2%), bone sarcoma (N=183, 1.8%), head and neck carcinoma (N=175, 1.7%), cancer of unknown primary (N=120, 1.2%), endocervical carcinoma (N=104, 1.0%), and others (N=412, 4.0%) . The median age of patients was 58 years old, ranging from ≤1 to 96 years old. In the cohort, 60.4% were male and 39.6% were female.
Recruitment	From 2017 to 2018, a total of 10,194 pathologically diagnosed solid tumor samples either resected or biopsied were collected from patients across all of China, including 4222 (41.4%) from East China, 2983 (29.3%) from South China, 966 (9.5%) from Southwest China, 953 (9.3%) from North China, 505 (5.0%) from Central China, 333 (3.3%) from Northwest China, and 232 (2.3%) from Northeast China.
Ethics oversight	This study was conducted according to the Declaration of Helsinki and approved by the Institutional Review Board of the Shandong Provincial Hospital and the Shanghai Ethics Committee for Clinical Research. All patients provided written informed consent.

Note that full information on the approval of the study protocol must also be provided in the manuscript.

Field-specific reporting

Please select the one below that is the best fit for your research. If you are not sure, read the appropriate sections before making your selection.

☒ Life sciences ☐ Behavioural & social sciences ☐ Ecological, evolutionary & environmental sciences

For a reference copy of the document with all sections, see nature.com/documents/nr-reporting-summary-flat.pdf

Life sciences study design

All studies must disclose on these points even when the disclosure is negative.

Sample size	Based on the 10,194 patients of this cohort, the genomic alterations of 40 patient with NTRK fusion were selected for further analysis.
Data exclusions	There are no other data exclusion criteria.
Replication	This is a retrospective study and does not involve any replications.
Randomization	This is a retrospective study and mainly focus on the NTRK fusion. Therefore, randomization is not relevant to our study.
Blinding	This is a retrospective study and mainly focus on the NTRK fusion. Therefore, blinding is not relevant to our study.

Reporting for specific materials, systems and methods

We require information from authors about some types of materials, experimental systems and methods used in many studies. Here, indicate whether each material, system or method listed is relevant to your study. If you are not sure if a list item applies to your research, read the appropriate section before selecting a response.

Materials & experimental systems

n/a	Involved in the study
☒	☐ Antibodies
☒	☐ Eukaryotic cell lines
☒	☐ Palaeontology and archaeology
☒	☐ Animals and other organisms
☒	☐ Clinical data
☒	☐ Dual use research of concern
☒	☐ Plants

Methods

n/a	Involved in the study
☒	☐ ChIP-seq
☒	☐ Flow cytometry
☒	☐ MRI-based neuroimaging
